# CAVER: a new tool to explore routes from protein clefts, pockets and cavities

**DOI:** 10.1186/1471-2105-7-316

**Published:** 2006-06-22

**Authors:** Martin Petřek, Michal Otyepka, Pavel Banáš, Pavlína Košinová, Jaroslav Koča, Jiří Damborský

**Affiliations:** 1 National Center for Biomolecular Research, Masaryk University, Kamenice 5/A4, 625 00 Brno, Czech Republic; 2 Department of Physical Chemistry and Center for Biomolecules and Complex Molecular Systems, Palacký University, tř. Svobody 26, 771 46 Olomouc, Czech Republic; 3 Loschmidt Laboratories, Masaryk University, Kamenice 5/A4, 625 00 Brno, Czech Republic

## Abstract

**Background:**

The main aim of this study was to develop and implement an algorithm for the rapid, accurate and automated identification of paths leading from buried protein clefts, pockets and cavities in dynamic and static protein structures to the outside solvent.

**Results:**

The algorithm to perform a skeleton search was based on a reciprocal distance function grid that was developed and implemented for the CAVER program. The program identifies and visualizes routes from the interior of the protein to the bulk solvent. CAVER was primarily developed for proteins, but the algorithm is sufficiently robust to allow the analysis of any molecular system, including nucleic acids or inorganic material. Calculations can be performed using discrete structures from crystallographic analysis and NMR experiments as well as with trajectories from molecular dynamics simulations. The fully functional program is available as a stand-alone version and as plug-in for the molecular modeling program PyMol. Additionally, selected functions are accessible in an online version.

**Conclusion:**

The algorithm developed automatically finds the path from a starting point located within the interior of a protein. The algorithm is sufficiently rapid and robust to enable routine analysis of molecular dynamics trajectories containing thousands of snapshots. The algorithm is based on reciprocal metrics and provides an easy method to find a centerline, i.e. the spine, of complicated objects such as a protein tunnel. It can also be applied to many other molecules. CAVER is freely available from the web site .

## Background

The shape of a protein is complicated by its many clefts, pockets, protrusions, channels and cavities. Protein concavities offer a unique microenvironment for biological functions, such as ligand binding or enzymatic catalysis. Protein shape is of great interest to medicinal chemists working in the drug discovery industry and looking for inhibitors, enzymologists interested in identifying substrate molecules based on the well known "lock and key" mechanism and protein chemists studying protein-protein or protein-DNA interactions. The identification of protein pockets and cavities has been the focus of several studies [1-4] and various algorithms have been developed for the calculation of protein volume and surface area. A large number of enzymes possess buried active sites that are connected to the external solvent environment by access routes (tunnels or channels). A catalytic step must always be preceded by the formation of an enzyme-substrate complex, which may require passage of the substrate through these routes. The size and shape of the access routes may become an important determinant of enzyme substrate specificity [5]. Changes in the diameter of the access tunnels during the dynamic movement of a protein play an important biological role, such as that described for acetylcholinesterase [6]. Two narrow active site gorges are positioned deep inside the protein core and movement of the residues making up the gorge walls is necessary to allow ligands access to the active site. A method based on molecular surface was used for the calculation of the gorge diameter in acetylcholinesterase. The diameter was defined as the maximum probe size that produces a continuous molecular surface between an active site and a solvent. Calculation of one diameter in this approach requires the generation of several molecular surfaces using a series of probes of increasing size [7]. A more effective method is implemented in the CAST program, which utilizes the alpha shape theory. CAST computation of pockets and their openings does not require direct human interaction. The required inputs are atomic coordinates, van der Waals radii, and the radius of the probe sphere [4]. The program VOIDOO, a component of O package utilizes a grid-based algorithm for detection, delineation, and measurement of protein cavities and solvent accessible pockets. The VOIDOO algorithm suffers from crude grid spacing and the "can-of-worms" phenomenon [1]. The central problem in the analysis of tunnels in protein structures is the identification of the centerline, i.e. spine, of a 3D object. Algorithms dealing with centerlines have been applied to medical procedures, for example in virtual colonoscopy and bronchoscopy [8-11].

The aim of this study was to develop a rapid and accurate algorithm for the identification of routes from buried active sites to the external solvent in static protein structures. We aimed to produce an algorithm that could also be applied to molecular dynamic trajectories. Further, the algorithm was intended to allow changes in the radius of a channel gorge with time to be monitored and the most probable access routes to be identified. Several other requirements were taken into consideration during development of the algorithm and its implementation: (i) speed, thus enabling rapid analysis of an entire trajectory from a molecular dynamic simulation, i.e. thousands of snapshots, in a few hours; (ii) easy identification of a starting point for the calculation; (iii) that the algorithm functions independently of the probe radius; (iv) storage of paths in PDB format; and (v) intuitive visualization.

## Implementation

### The algorithm

**Figure 1:**
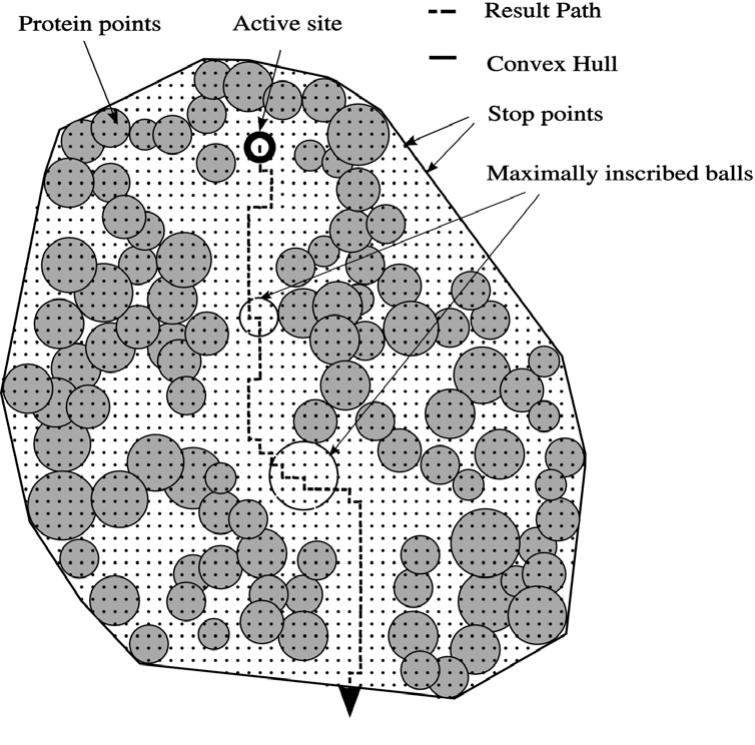
**Sketch of the method implemented in CAVER**. The black bold circle represents the starting point. The protein is visualized by gray circles with van der Walls atom radii mapped on a discrete grid (black dots). The solid line represents the boundary between the protein (convex hull) interior and its surroundings. Empty circles represent the maximally inscribed balls on the probable route (dashed line).

The most accessible path from the protein cavity to the bulk solvent has to be found by systematic exploration of the protein interior, in order to calculate the access route gorge radius (Fig. 1). In our model, a protein consists of hard sphere atoms with appropriate van der Waals radii. The protein body is modeled on a discrete three-dimensional grid space and all grid nodes are clustered into two classes: nodes located in the interior of the protein body (inside atomic vdW radii) and nodes located outside the protein body. Outer nodes can lie in the cavity, access tunnels or in the external environment of the protein, e.g. a bulk solvent. The convex approximation of the protein, termed the 'convex hull', is used to distinguish nodes that lie either in the interior or exterior of the protein (Fig. 1). Nodes that are located outside of the convex hull, are eliminated and not used in further calculations.

**Figure 2:**
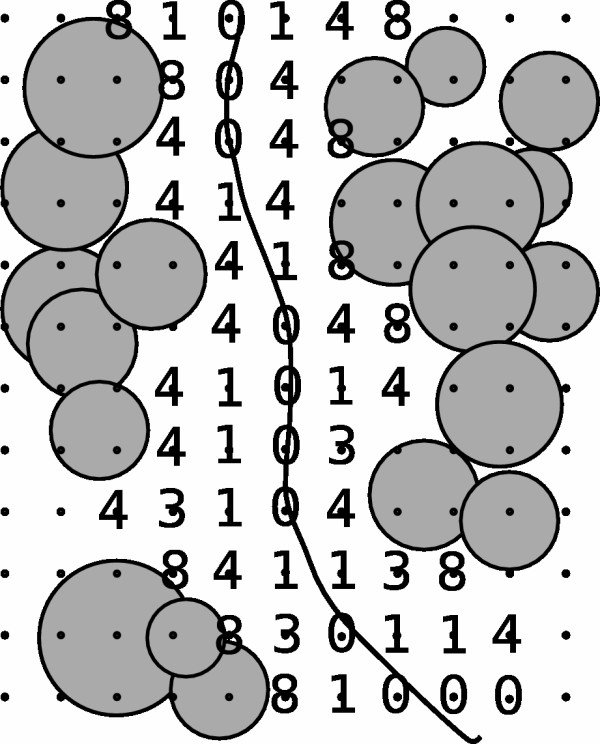
**Evaluation of grid nodes by cost function**. A grid point evaluation using the cost function (Eq. 3). The line represents the optimal centerline.

Attention is paid to nodes that lie on a boundary of the modeled convex hull. These nodes are potential end-stops of the grid search algorithm because each boundary node can be treated as a putative outfall of the channel. The mathematical object, which is called a vertex-weighted graph, is constructed and the algorithm applied to identify the shortest low-cost path. Each possible path from the active site to the exterior is evaluated as a positive value. This value represents the relative cost to navigate each path in what could be described as a "highway-toll". Long and complicated paths are more "expensive", while the short direct paths are "cheaper" (Fig. 2). More formally, the cost function *C(P) *is defined (Eq. 1) for the given path *P *as the sum of node-price-function values calculated for all nodes forming the path *P*. Let *N(P) *be the set of all points form the path *P*, then the *C(P) *is expressed as

C(P)=∑x∈N(P)c(x).     (1)
 MathType@MTEF@5@5@+=feaafiart1ev1aaatCvAUfKttLearuWrP9MDH5MBPbIqV92AaeXatLxBI9gBaebbnrfifHhDYfgasaacH8akY=wiFfYdH8Gipec8Eeeu0xXdbba9frFj0=OqFfea0dXdd9vqai=hGuQ8kuc9pgc9s8qqaq=dirpe0xb9q8qiLsFr0=vr0=vr0dc8meaabaqaciaacaGaaeqabaqabeGadaaakeaacqWGdbWqcqGGOaakcqWGqbaucqGGPaqkcqGH9aqpdaaeqbqaaiabdogaJjabcIcaOiabdIha4jabcMcaPaWcbaGaemiEaGNaeyicI4SaemOta4KaeiikaGIaemiuaaLaeiykaKcabeqdcqGHris5aOGaeiOla4IaaCzcaiaaxMaadaqadaqaaiabigdaXaGaayjkaiaawMcaaaaa@43E1@

This cost function depends on the number of nodes in each sum and, as such, this function is not suitable for the purposes of comparison. A normalized cost function is defined (Eq. 2) to avoid cost function dependence on a summand number:

Cn(P)=1N∑x∈N(P)c(x),     (2)
 MathType@MTEF@5@5@+=feaafiart1ev1aaatCvAUfKttLearuWrP9MDH5MBPbIqV92AaeXatLxBI9gBaebbnrfifHhDYfgasaacH8akY=wiFfYdH8Gipec8Eeeu0xXdbba9frFj0=OqFfea0dXdd9vqai=hGuQ8kuc9pgc9s8qqaq=dirpe0xb9q8qiLsFr0=vr0=vr0dc8meaabaqaciaacaGaaeqabaqabeGadaaakeaacqWGdbWqdaWgaaWcbaGaemOBa4gabeaakiabcIcaOiabdcfaqjabcMcaPiabg2da9maalaaabaGaeGymaedabaGaemOta4eaamaaqafabaGaem4yamMaeiikaGIaemiEaGNaeiykaKcaleaacqWG4baEcqGHiiIZcqWGobGtcqGGOaakcqWGqbaucqGGPaqkaeqaniabggHiLdGccqGGSaalcaWLjaGaaCzcamaabmaabaGaeGOmaidacaGLOaGaayzkaaaaaa@479F@

where N is the number of the summand. Next, the single node cost function *c(x) *must fulfill two requirements. First, it must provide a positive value for each node and a low-value for nodes that are surrounded by empty space. Second, it must identify preferred nodes that are surrounded by sufficient empty space to allow a hypothetical substrate to pass through a channel without risk of collision. These low-weighted nodes are preferentially selected by the search-algorithm. In our case, the cost function *c(x) *for a single node was chosen (Eq. 3)

c(x)=1(rmax⁡(x)+ε)2,     (3)
 MathType@MTEF@5@5@+=feaafiart1ev1aaatCvAUfKttLearuWrP9MDH5MBPbIqV92AaeXatLxBI9gBaebbnrfifHhDYfgasaacH8akY=wiFfYdH8Gipec8Eeeu0xXdbba9frFj0=OqFfea0dXdd9vqai=hGuQ8kuc9pgc9s8qqaq=dirpe0xb9q8qiLsFr0=vr0=vr0dc8meaabaqaciaacaGaaeqabaqabeGadaaakeaacqWGJbWycqGGOaakcqWG4baEcqGGPaqkcqGH9aqpdaWcaaqaaiabigdaXaqaaiabcIcaOiabdkhaYnaaBaaaleaacyGGTbqBcqGGHbqycqGG4baEaeqaaOGaeiikaGIaemiEaGNaeiykaKIaey4kaSccciGae8xTduMaeiykaKYaaWbaaSqabeaacqaIYaGmaaaaaOGaeiilaWIaaCzcaiaaxMaadaqadaqaaiabiodaZaGaayjkaiaawMcaaaaa@462C@

where function *r*_max _*(x) *is equal to the maximal radius of a hypothetical ball that can be inserted into node x just touching the protein surface. The small constant *ε *is here only for technical purposes to get rid of a singularity of the function in points where *r*_max _*(x) *equals zero.

**Figure 3:**
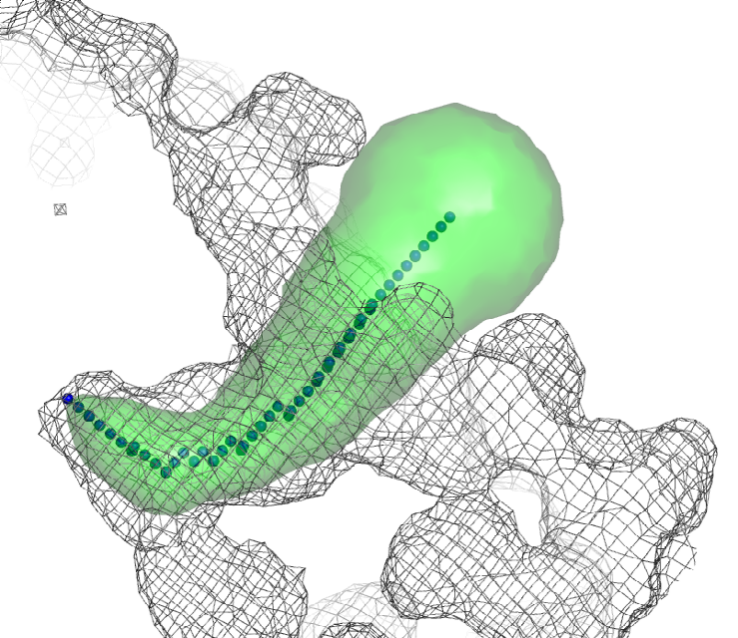
**Access path visualized by pyMol**. Visualization of the access route using the PyMol plug-in. Wires represent the protein surface, balls are nodes and the surface represents the export route.

**Figure 4:**
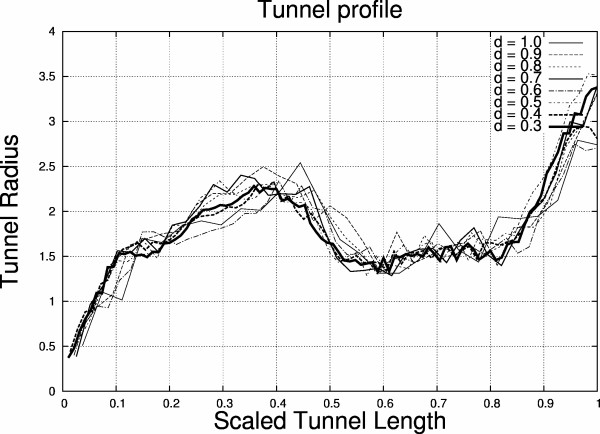
**Path profile convergence**. Convergence of the path profile found on the grid with increasing precision, i.e. decreasing node distance *d*. Calculation of one path takes approximately 11 sec in case of *d *= 0.7°A (Athlon 2600+, 2GB RAM, NetBSD 1.6.1) but increases ten-fold in a substrate where *d *= 0.3°A.

The graph-searching algorithm then establishes the lowest cost path from the active site to the external environment. The calculated path can be visualized (Fig. 3 and Fig. 4) using the *r*_max _radii for each node of the path. The smallest radius represents the channel gorge and as such the point coordinates can be determined together with the gorge radius *r*_gorge_.

### Implementation details

The method was implemented in the CAVER program (Additional file 1 and web page . The program uses the publicly-licensed software qhull [12] that quickly (in *O(N log N) *time) computes a convex hull for a given set of *N *points in three dimensions. The result of qhull was used to eliminate nodes located outside the convex hull. All points were investigated regardless of whether they lay inside or outside the convex hull. This can be achieved by traversing all facets of the convex hull and testing whether a point lies in the same halfspace as another convex hull point. This process takes *O(NK) *time, where *N *is the number of points inside the convex hull, and *K *is the number of facets forming the convex hull.

Based on graph theory, several methods for the shortest path problem have been described. The most widely used algorithms are Dijkstra's [13], Bellman-Ford's [14,15], A* search algorithm, and the Floyd-Warshall algorithm [16]. In our case, a positively vertex-weighted graph is plotted on a three dimensional grid, where the source vertex is known while the destination vertex is not. We used a modified form of the Dijkstra's algorithm.

The Dijkstra's algorithm effectively solves the problem of the shortest path from a single source vertex to the destination one. It was originally designed for edge-weighted graphs but its vertex-weighted variation is easy to implement. The algorithm can be used even if the destination vertex is unknown. In the main loop of the algorithm, the shortest path to the closest available vertex (measured from the source vertex) is determined. Then, estimates of the shortest path for all the adjacent vertices are updated. This means searching can be terminated if the nearest available vertex is the boundary vertex indicating that the shortest path has been identified. To speed up the algorithm, a modification related to the cost function was implemented. The single node cost function is evaluated as part of the main loop of the Dijkstra's algorithm. The cost function is evaluated only at nodes where it is required.

The identified path can be easily visualized since the program writes a PDB file containing the coordinates of the path nodes accompanied by the maximum probe radius that touches the vdW protein surface (Fig. 3). A user-friendly GUI was implemented in the graphic program PyMol [17].

### Method performance

An algorithm to perform a skeleton search on a defined grid was developed and implemented in the CAVER program as described in the Methods section. The algorithm developed automatically finds the easiest path from the starting point, typically located inside the molecule, to the exterior of the molecule. The identified path resembles a tunnel that connects protein clefts, pockets or cavities with the surrounding bulk solvent. The tunnel characteristics, e.g. length, mean radius and gorge radius are determined and can be further analyzed. In molecular dynamics trajectories it is possible to analyze time fluctuations of tunnel characteristics and construct a dynamic picture of tunnel behavior.

The tunnel gorge radius *r*_gorge _is one of the most important tunnel characteristics because the tunnel gorge can form a bottleneck for substrate access or product release to and from the active site of a protein. The radius *r*_gorge _as estimated by the algorithm is always underestimated in finite grids. The maximal error "max" of an *r*_gorge _estimation is expressed by the equation (Eq. 4):

εmax⁡(rgorge)=32d,     (4)
 MathType@MTEF@5@5@+=feaafiart1ev1aaatCvAUfKttLearuWrP9MDH5MBPbIqV92AaeXatLxBI9gBaebbnrfifHhDYfgasaacH8akY=wiFfYdH8Gipec8Eeeu0xXdbba9frFj0=OqFfea0dXdd9vqai=hGuQ8kuc9pgc9s8qqaq=dirpe0xb9q8qiLsFr0=vr0=vr0dc8meaabaqaciaacaGaaeqabaqabeGadaaakeaaiiGacqWF1oqzdaWgaaWcbaGagiyBa0MaeiyyaeMaeiiEaGhabeaakiabcIcaOiabdkhaYnaaBaaaleaacqWGNbWzcqWGVbWBcqWGYbGCcqWGNbWzcqWGLbqzaeqaaOGaeiykaKIaeyypa0ZaaSaaaeaadaGcaaqaaiabiodaZaWcbeaaaOqaaiabikdaYaaacqWGKbazcqGGSaalcaWLjaGaaCzcamaabmaabaGaeGinaqdacaGLOaGaayzkaaaaaa@45F5@

where *d *is equal to the length of the grid cell edge. The probability of ε_max _realization is equal to zero and this error is overestimated, therefore the mean error should be defined. The mean error of *r*_gorge _determination is equal to (Eq. 5):

〈εmax⁡(rgorge)〉=1V∭d3rdV≅0.48d,     (5)
 MathType@MTEF@5@5@+=feaafiart1ev1aaatCvAUfKttLearuWrP9MDH5MBPbIqV92AaeXatLxBI9gBaebbnrfifHhDYfgasaacH8akY=wiFfYdH8Gipec8Eeeu0xXdbba9frFj0=OqFfea0dXdd9vqai=hGuQ8kuc9pgc9s8qqaq=dirpe0xb9q8qiLsFr0=vr0=vr0dc8meaabaqaciaacaGaaeqabaqabeGadaaakeaacqGHPms4iiGacqWF1oqzdaWgaaWcbaGagiyBa0MaeiyyaeMaeiiEaGhabeaakiabcIcaOiabdkhaYnaaBaaaleaacqWGNbWzcqWGVbWBcqWGYbGCcqWGNbWzcqWGLbqzaeqaaOGaeiykaKIaeyOkJeVaeyypa0ZaaSaaaeaacqaIXaqmaeaacqWGwbGvaaWaa8WuaeaacqWGYbGCcqWGKbazcqWGwbGvaSqaaiabdsgaKnaaCaaameqabaGaeG4mamdaaaWcbeqdcqGHRiI8cqGHRiI8cqGHRiI8aOGaeyyrIaKaeGimaaJaeiOla4IaeGinaqJaeGioaGJaemizaqMaeiilaWIaaCzcaiaaxMaadaqadaqaaiabiwda1aGaayjkaiaawMcaaaaa@5AEA@

and its variance and deviation equal to (Eq. 6)

var⁡(ε)=〈εmax⁡2(rgorge)〉−〈εmax⁡(rgorge)〉2var⁡(ε)≅0.019d2,σε≅0.14d,     (6)
 MathType@MTEF@5@5@+=feaafiart1ev1aaatCvAUfKttLearuWrP9MDH5MBPbIqV92AaeXatLxBI9gBaebbnrfifHhDYfgasaacH8akY=wiFfYdH8Gipec8Eeeu0xXdbba9frFj0=OqFfea0dXdd9vqai=hGuQ8kuc9pgc9s8qqaq=dirpe0xb9q8qiLsFr0=vr0=vr0dc8meaabaqaciaacaGaaeqabaqabeGadaaakeaafaqadeGabaaabaGagiODayNaeiyyaeMaeiOCaiNaeiikaGccciGae8xTduMaeiykaKIaeyypa0JaeyykJeUae8xTdu2aa0baaSqaaiGbc2gaTjabcggaHjabcIha4bqaaiabikdaYaaakiabcIcaOiabdkhaYnaaBaaaleaacqWGNbWzcqWGVbWBcqWGYbGCcqWGNbWzcqWGLbqzaeqaaOGaeiykaKIaeyOkJeVaeyOeI0IaeyykJeUae8xTdu2aaSbaaSqaaiGbc2gaTjabcggaHjabcIha4bqabaGccqGGOaakcqWGYbGCdaWgaaWcbaGaem4zaCMaem4Ba8MaemOCaiNaem4zaCMaemyzaugabeaakiabcMcaPiabgQYiXpaaCaaaleqabaGaeGOmaidaaaGcbaGagiODayNaeiyyaeMaeiOCaiNaeiikaGIae8xTduMaeiykaKIaeyyrIaKaeGimaaJaeiOla4IaeGimaaJaeGymaeJaeGyoaKJaemizaq2aaWbaaSqabeaacqaIYaGmaaGccqGGSaalcqWFdpWCdaWgaaWcbaGae8xTdugabeaakiabgwKiajabicdaWiabc6caUiabigdaXiabisda0iabdsgaKjabcYcaSaaacaWLjaGaaCzcamaabmaabaGaeGOnaydacaGLOaGaayzkaaaaaa@7EE2@

The *r*_gorge _estimation should be corrected by adding 0.48*d *to the *r*_gorge_. The corrected *r*_gorge _estimate has a mean error value μ_ε _= 0 and the variance of error is var(ε) = 0.019*d*^2^. In the case of a globular protein (50 × 50 × 50 Å^3^) the ε_max _(*r*_gorge_) costs 0.43 Å for a grid with *d *= 0.5 Å, however, the mean ε(*r*_gorge_) equals 0.24 Å, and σ_ε _= 0.07 Å. The results of the tests (depicted in Fig. 4) focus on the convergence of the identified paths with *d *decreasing from 1.0 to 0.3 Å.

Performance of the method is given as the tunnel volume i.e. number of vertexes searched rather than the number of atoms. In the case of haloalkane dehalogenases (the active site volume ~200Å^3^) the typical calculation of one tunnel takes about 10–12 sec. but in the case of cytochrome P450 2C9 or 3A4 (which has larger active sites ~500–600Å^3^) the calculation takes about 20–25 sec. In case of very large cavities (e.g. RNA) calculation may take several minutes at low resolution (*d *= 1–2Å). The program performance was tested on Pentium IV 3.2 GHz machine with 2 GB RAM running on Windows XP Professional operating system.

## Results

### Case study

**Figure 5:**
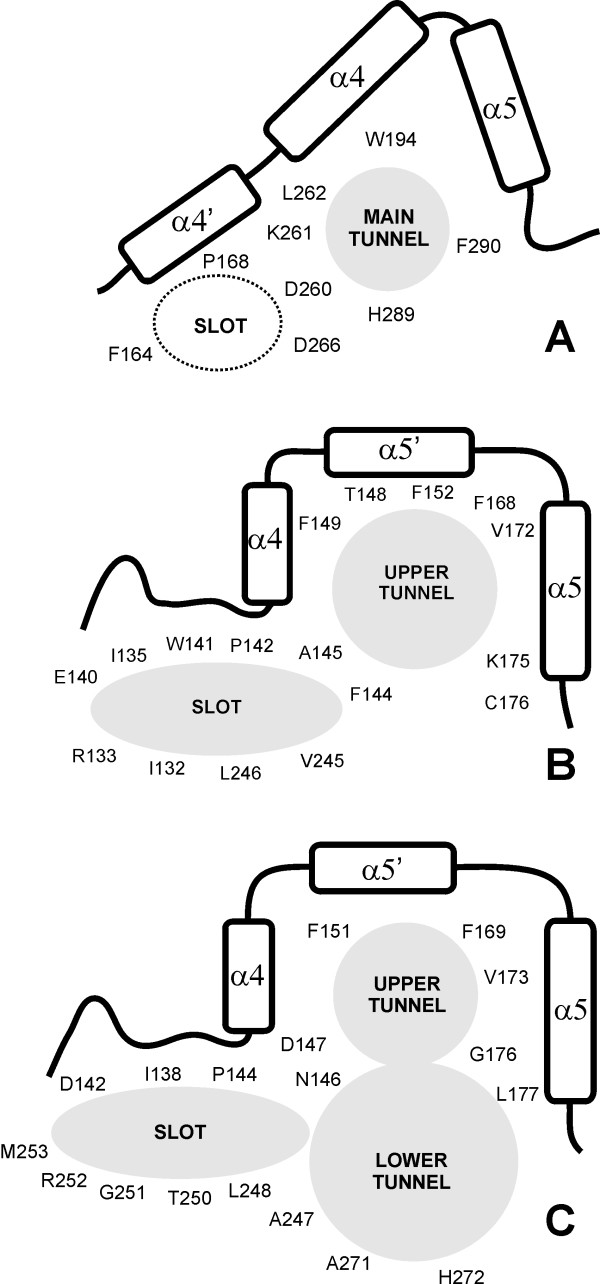
**Scheme of haloalkane dehalogenases' tunnels**. Schematic representation of access paths for DhlA (A), DhaA (B) and LinB (C) identified by protein crystallography [19–31] and molecular dynamic simulations [32]. The slot in DhlA was described in this study.

Haloalkane dehalogenases (EC 3.8.1.5) are microbial enzymes that cleave a carbon-halogen bond in a broad range of halogenated compounds [18]. The molecular structures of three different haloalkane dehalogenases are known: DhlA from *Xanthobacter autotrophicus *GJ10 [19-26], DhaA from *Rhodococcus *sp. [27] and LinB from *Sphingomonas paucimobilis *UT26 [28-31]. The overall shape of haloalkane dehalogenases is globular, with the active site buried between the main domain of an α/β hydrolase fold and a cap domain with a uteroglobin fold. There are three access routes connecting the protein surface with the active site, denoted the main tunnel, the upper tunnel and a slot (Fig. 5). The three proteins differ in the number of routes that provide access to their active site: LinB has the most available active site, accessible through three tunnels. The active site in DhaA is accessible through the upper tunnel and the slot, while DhlA is believed to have a single accessible route via the main tunnel [32]. Here we used CAVER to conduct a thorough analysis of the access paths using all available X-ray structures and pre-calculated molecular dynamics trajectories.

### Analysis of X-ray structures

**Figure 6:**
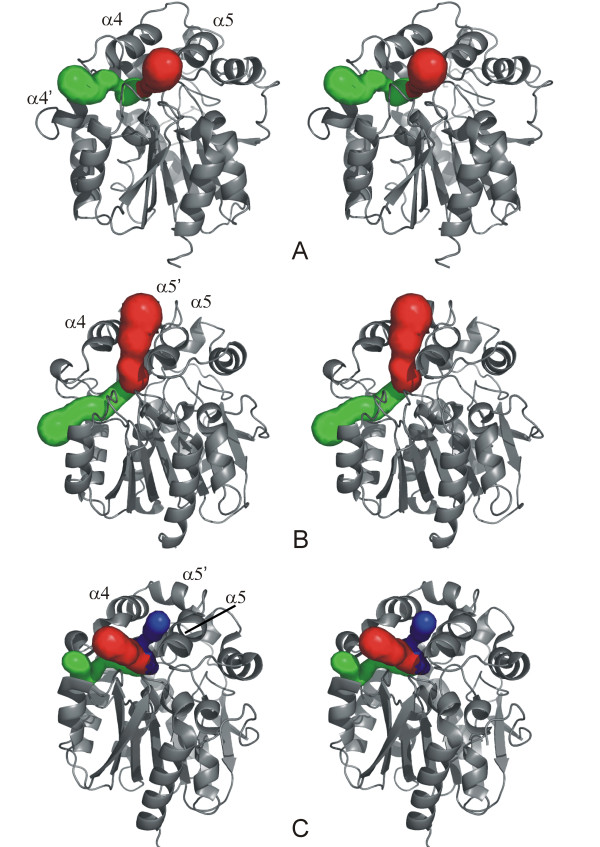
**Tunnels found by CAVER**. Accessible paths identified by CAVER in DhlA (A), DhaA (B) and LinB (C). The most accessible tunnel in every structure (main in DhlA, upper in DhaA and lower in LinB) is colored in red. The upper tunnel of LinB is in blue. Slots are highlighted in green for each structure.

Analysis of fifteen available crystal structures of the DhlA enzyme (1B6G, 1BE0, 1BEE, 1BEZ, 1CIJ, 1EDB, 1EDD, 1EDE, 1HDE, 2DHC, 2DHD, 2DHE, 2HAD, 2EDA, and 2EDC) revealed two similar access routes (Fig. 6A). The main tunnel was identified as the most accessible route in nine of the structures (underlined in the list above). In six structures, the most accessible tunnel was equivalent to the slot in DhaA and LinB. The gorge, i.e. the bottleneck, of the main tunnel is made up of W175, L262 and H289, the gorge of the slot is formed by P168, F164, F172 and the backbone by G171, K261 and L262. In the next step, a more systematic analysis was conducted to identify additional paths. Four paths were calculated for each structure and averaged (Table 1). CAVER found that, in each case, two tunnels were equivalent to previously described paths, i.e. the main tunnel and the slot. Two other paths had significantly higher cost function and a narrower gorge (Table 1), making it less likely that they fulfill a biological role. We note that the crystallographic analysis [22] revealed one access tunnel, while two parallel access paths were deduced from the kinetic data [26]. The existence of the second tunnel in DhlA provides an additional explanation for the elevated activity of F172W with 1-chlorohexane, which is attributed to the increased flexibility of the 'helix-loop-helix' region [26].

The three crystal structures available for the DhaA enzyme (1BN6, 1BN7 and 1CQW) were analyzed for the presence of access routes. CAVER detected one clearly preferred route, which corresponded to the upper tunnel. The gorge of this tunnel is made up of W141, F144, A145, F159, V172 and C176 (numbered according to *dhaA *sequence). The additional paths were located in the slot (Fig. 6B). The slots of the three structures studied showed a slightly different spatial position and variable size with respect to the mean gorge radius, mainly due to repositioning of the side-chain R133. The cost function of these routes was almost twice that of the upper tunnel (Table 1), but still comparable with the main tunnel and the slot of DhlA, which are known to be involved in substrate binding and product release. Exchange of solvent molecules between the active site and the slot was observed during a 1ns molecular dynamic simulation [32].

Analysis of eleven available LinB crystal structures (1CV2, 1D07, 1K5P, 1K6E, 1MJ5, 1K63, 1IZ8, 1IZ7, 1G5F, 1G4H and 1G42) identified the lower tunnel as the most accessible access route in nine of them (underlined). The gorge of this tunnel is made up of P144, D147, L177 and A247. The upper tunnel was identified as the most accessible route in two out of the nine structures. The gorge of the upper tunnel is formed by D147, F151, V173 and L177. Systematic searches of the four tunnels in the structures revealed the slot as another possible access route (Fig. 6C). The cost function of this route is, however, twice that of the lower tunnel (Table 1). Its gorge is formed by D142, P144, L248, G251 and M253. Previous molecular dynamics simulations [32] demonstrated that all three access routes can be used in the exchange of water molecules between the active site and the bulk solvent. The existence of alternative export routes explains the activity of LinB mutants that carry a large amino acid residue at position L177 [5]. L177 is located inside the lower tunnel and its substitution may result in closure of this tunnel.

### Analysis of molecular dynamics trajectories

**Figure 7:**
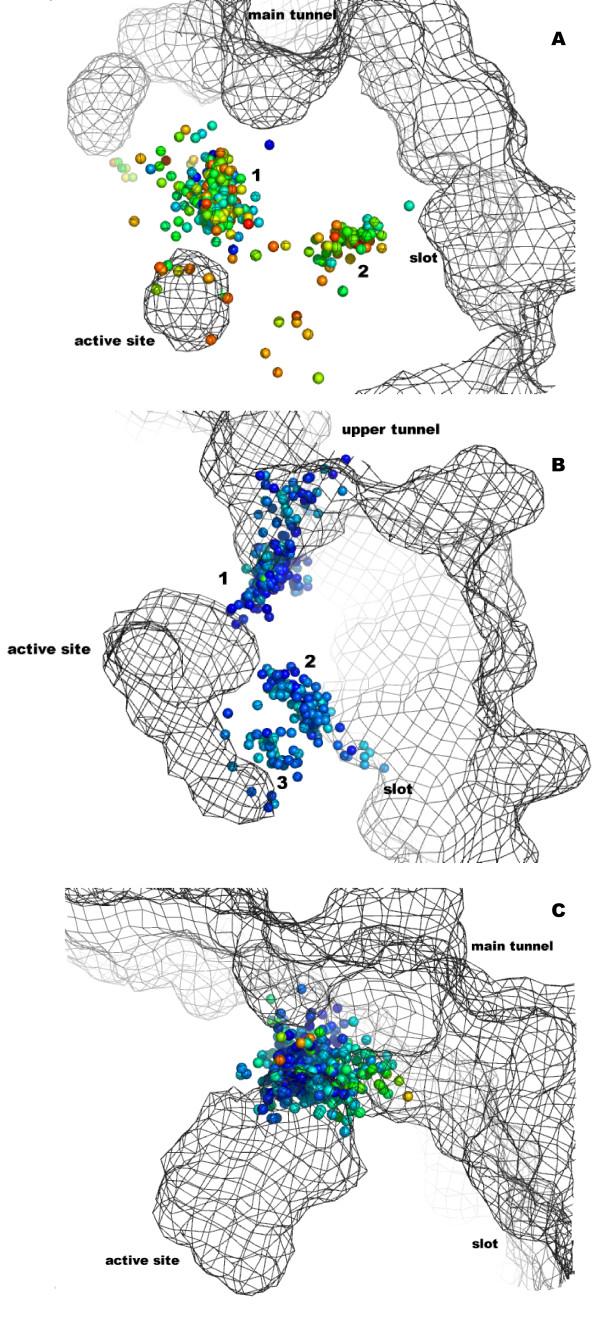
**Tunnels' gorges as found by CAVER in MD trajectories**. Analysis of snapshots taken from a molecular dynamics simulation of DhlA (A), DhaA (B) and LinB (C). Two clusters of tunnel gorges were identified in DhlA simulation (denoted by the numbers 1 and 2). Three clusters were identified in the DhaA simulation (denoted by the numbers 1–3) and one cluster was identified in the LinB simulation. Tunnel gorges are represented by small balls. Ball color correlates with gorge radius, balls representing narrow gorges are red and wide gorges are blue. The mesh represents the protein surface.

A molecular dynamics simulation of the DhlA enzyme was analyzed using CAVER to determine the easiest routes from the active site. About 400 snapshots taken at 5 ps intervals were analyzed, the tunnels identified and their corresponding gorgeswere further analyzed by visual inspection (Fig. 7A). CAVER identified two clusters of gorges that correspond to two different paths. The most populated tunnel gorges (78%) are located in the main tunnel and the other remaining gorge clusters are located in the slot.

As in the previous case, the molecular dynamics simulation of the DhaA enzyme was analyzed using the CAVER program. Two main clusters were identified by CAVER, one having two subclusters (Fig. 7B) of gorges resulting in three different paths,. The most populated access gorges (64%) were located in the upper tunnel and the two remaining gorge clusters were positioned in the slot. The two subclusters in the slot (Fig. 7B) have populations equal to 26% (cluster 2) and 10% (cluster 3), respectively. Analysis of the LinB molecular dynamics trajectory identifies the main tunnel as the most accessible route to the active site (Fig. 7C).

## Conclusion

A new algorithm for the identification of tunnels in large molecular systems was developed and implemented in the CAVER program, which is available within the public domain. The algorithm automatically explores a grid, which is constructed over the molecule and stripped to its convex hull. Nodes are evaluated using a cost function, which determines the amount of free space around the node. The grid search algorithm is used to find the lowest-cost centerline path between a given starting point and the surface of the molecule. The user needs only to provide the molecular geometry, atomic van der Waals radii and the designated starting point, to enable the analysis of any molecular system be it protein, nucleic acid or inorganic material. The algorithm is sufficiently rapid and robust for the routine analysis of molecular dynamics trajectories that contain thousands of snapshots. The program is also available as a plug-in for PyMol and, additionally a Web-based version of the program offers analysis of static protein structures online.

## Availability and requirements

Project name: CAVER;

Project home page: ;

Operating systems: UNIX/Linux, Windows;

Programming language: C++;

Other requirements: Qhull package required 

Licence: CAVER licence;

Any restrictions to use by non-academics: licence needed.

## Authors' contributions

MP developed the algorithm for search of access paths, wrote and tested the software, prepared initial draft of the manuscript and prepared web pages; MO developed concept of the software, conducted performance tests, wrote parts of the manuscript and prepared graphic material for the web; PB contributed ideas on algorithm and conducted statistical analyses; PK conducted performance tests; JK financially supported MP; JD contributed fundamental biochemical concept and interpreted data, wrote parts of the manuscript, contributed ideas on web pages and coordinated project.

## Supplementary Material

Additional File 1 **Source codes of CAVER**. Additional file (gzipped tar archive caver_unix_v0.99.4.tar.gz) contains source codes of CAVER ver. 0.99.4 for UNIX platforms and pyMol plug-in. Instructions for the installation and updates are provided at web page . Click here for file

## Figures and Tables

**Table 1:** Characteristics of tunnels. Characteristics of the four most accessible paths connecting the active sites of DhlA, DhaA and LinB with the bulk solvent.

protein	paths^a^	r_gorge _[Å]	*C*	l [Å]
DhlA	slot	0.89 ± 0.04	34.2 ± 2.6	16.1 ± 0.4
DhlA	main tunnel	0.86 ± 0.05	34.8 ± 4.7	15.1 ± 0.4
DhlA	unassigned	0.71 ± 0.08	46.2 ± 6.1	15.6 ± 0.3
DhlA	unassigned	0.70 ± 0.11	51.6 ± 12.1	16.6 ± 1.5

DhaA	upper tunnel	1.47 ± 0.16	14.3 ± 7.7	13.8 ± 0.6
DhaA	slot	1.28 ± 1.02	24.0 ± 9.4	15.0 ± 4.3
DhaA	slot	1.00 ± 0.16	29.0 ± 10.7	15.5 ± 3.9
DhaA	slot	0.97 ± 0.35	32.0 ± 16.4	14.8 ± 2.4

LinB	lower tunnel	1.41 ± 0.11	16.3 ± 1.6	14.2 ± 0.7
LinB	upper tunnel	1.11 ± 0.15	22.6 ± 6.8	14.1 ± 0.6
LinB	slot	0.97 ± 0.08	31.7 ± 4.7	15.1 ± 0.4
LinB	slot	0.83 ± 0.06	35.4 ± 5.8	14.7 ± 0.7

^a^annotation of paths is provided in Figure 5, r_rgorge _– gorge radius; C – mean cost function; l – mean length of the tunnel. The values are averaged over all available X-ray structures.
